# Umbilical Cord Milking: A Review

**DOI:** 10.3389/fped.2018.00335

**Published:** 2018-11-13

**Authors:** Anup C. Katheria

**Affiliations:** ^1^Sharp Mary Birch Hospital for Women & Newborns, San Diego, CA, United States; ^2^Loma Linda University, Loma Linda, CA, United States

**Keywords:** placental transfusion, umbilical cord milking, delayed cord clamping, resuscitation, neonates

## Abstract

This is a review of umbilical cord milking, a controversial technique where the umbilical cord is squeezed several times before it is clamped an cut. While not physiological or natural for newborns, the question lies as to whether it is useful in certain circumstances, namely the depressed newborn. Here we review the literature and discuss why it could be considered as an alternative for the current practice of delayed cord clamping.

## Umbilical cord milking: a review

Over the past decade, there has been a growing evidence that delayed cord clamping is beneficial in term and preterm newborns (1, 2). Meanwhile there is uncertainty about the best method of cord management for infants that are deemed by a provider to be too unstable or to require resuscitation. Delayed cord clamping (DCC) is defined as waiting at least 30–60 s before clamping the umbilical cord (3, 4). This time delay precludes some of the most premature or sickest infants from receiving this beneficial treatment. This has borne out in a number of randomized controlled trials where as many as a quarter of the subjects do not receive DCC (5, 6). Another technique, umbilical cord milking (UCM), consists of gently grasping the uncut umbilical cord and squeezing the cord from the placenta several times toward the infant. In contrast to delayed cord clamping, milking provides a placental transfusion without postponing resuscitation and can be completed as quickly as immediate cord clamping.

The available studies comparing UCM to ICC in term infants include one systematic review (7), five RCTs (8–12), and five older controlled trials (13–17). These studies conclude in aggregate that cord milking significantly improves blood pressure, hematocrit, and hemoglobin levels within the first few days of life and iron stores out to 6 months of age. No associated harm was identified in any study.

For preterm infants, a systematic review (7) and 10 RCTs (18–26) comparing UCM to ICC demonstrate increased blood pressure, hemoglobin, urine output, cerebral oxygenation, decreased risk of intraventricular hemorrhage (IVH) of all grades, lower chronic lung disease (defined as oxygen requirement at 36 weeks), less necrotizing enterocolitis, lower levels of circulating cytokines and reduced need for transfusions. In late preterm infants, higher ferritin levels at 6 weeks of age have been reported after UCM (27). None of the studies demonstrated harm from UCM in these vulnerable infants, and provide strong evidence that cord milking effectively accelerates placental transfusion at birth resulting in benefits superior to ICC.

To date there are only two trials comparing UCM to DCC in premature infants (28, 29). Rabe et al. demonstrated a similar placental transfusion with a 30-second delay compared to milking the intact umbilical cord four times. Our group demonstrated that infants born by Cesarean Section had improved systemic blood flow (measured by echocardiography), blood pressure, hemoglobin levels and urine output in the first 72 h of life suggested an improved placental transfusion in this subgroup (30). This is significant since 60–70 percent of premature and emergent deliveries are by Cesarean section (31). We speculate that more blood remains in the placenta when a neonate is delivered by Cesarean Section because the anesthetic and surgical interventions interfere with the active contraction of the uterine muscles to expel the placenta.

## Breathing and cord clamping

**Figure 1:**
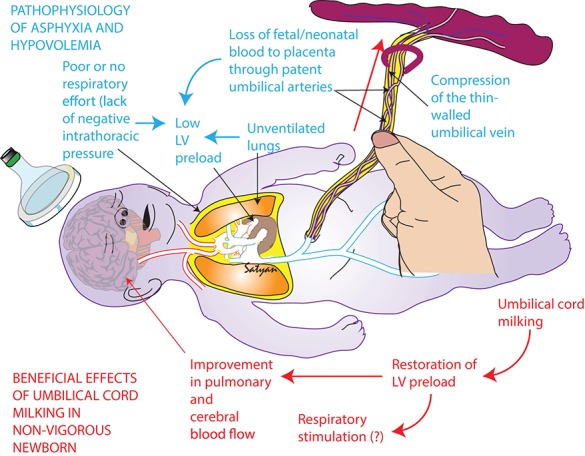
Beneficial effects of umbilical cord milking in non-vigorous newborns (Satyan Lakshminrusimha, UC Davis, copyright).

It has been suggested that waiting until the infant breathes before the cord is clamped could improve clinical outcomes (32). Animal studies have demonstrated that a physiologic based approach to clamp the cord when the infant has established breathing would be ideal (33). However, this does not preclude the infant from receiving UCM. In fact, UCM before clamping improves the pulmonary blood flow immediately at birth and assists lung expansion at the onset of respirations (see Figure 1, courtesy of S. Lakshminrusimha). Our previous trial of cord milking compared to immediate cord clamping demonstrated increased heart rate, oxygen saturation within the first 5 min of birth compared to immediate cord clamping (34). It also demonstrated decreased number of days on oxygen therapy and reduced chronic lung disease (21). The increase in blood volume to the lungs associated with cord milking has also been documented with recording of electrocardiographic changes; infants who had cord stripping had a longer P wave, PR, and QTC interval suggesting an increased right preload when compared with infants who had early clamping of the cord (17). This may explain why milking may promote earlier onset of breathing compared with DCC. In a pilot study comparing 60 s of delay with milking of the intact cord 4 times, more infants breathed before cord clamping with UCM compared with DCC (74 vs. 53%; 30. While repeat cord milking may allow some back flow of blood toward the placenta via the umbilical arteries, this also allows the afterload of the left ventricle to remain low while blood is being infused after each milking. The majority of cord milking trials have employed milking the cord before clamping which have demonstrated benefits in blood pressure, IVH, BPD and death (7).

## Cord management in infants that need resuscitation

Neonates requiring resuscitation may benefit from more blood returning to the body immediately after birth. It has been suggested that fetal blood volume loss to the placenta may occur as the infant descends into the birth canal when shoulder dystocia or with a tight nuchal cord occur (35, 36). This may be related to the squeeze of the infant as it traverses the tight birth canal, which places pressure on the umbilical cord. Within the cord, the muscular-walled, high-pressure arteries continue to move blood from the fetus to the placenta, while return flow from the placenta to the fetus in the thin-walled vein is diminished. This results in a net transfer of blood volume from the fetus to the placenta during birth (35).

The umbilical cord management currently recommended for infants that are non-vigorous (limp, pale and not breathing) and/or need resuscitation at birth is to immediately clamp (ICC) the umbilical cord (37). When ICC occurs, approximately 20 to 40 percent of the fetal-placental blood volume remains in the placenta (38, 39). This significant volume of fetal blood left in the placenta after ICC may further compromise neonatal transition and cardiac output resulting in lowered cerebral blood flow and tissue hypoxia, which can contribute to brain ischemia, multi-organ damage, or death (38, 40, 41). Immediate clamping may also be associated with bradycardia, increased pulmonary artery pressure with resulting right to left shunt, decreased in cardiac output, a surge in carotid artery pressure, all of which may contribute to increased NICU morbidity and mortality (32, 42).

The lack of studies in non-vigorous newborns has been identified as a major knowledge gap by the American Congress of Obstetricians & Gynecologists (ACOG), which states, “infants requiring resuscitation may benefit considerably from placental transfusion, but their need for immediate attention raises questions about whether they should undergo immediate or delayed umbilical cord clamping and whether umbilical cord milking (UCM) may offer a unique benefit” (43). UCM provides a replacement cardiac preload before the placenta is removed from the circulation and increases blood volume, which may improve cardiac output and increase pulmonary and cerebral circulation, thus mitigating further ischemia in an already compromised infant (16). For infants requiring immediate resuscitation at birth, neither of the methods currently practiced in all other infants to facilitating a placental transfusion, UCM or DCC, are recommended (43).

## Ventilation during delayed cord clamping

While several studies have demonstrated that resuscitating infants with an intact cord is feasible, much more research, training, pre-planning, and coordination are necessary as DCC is a challenge to perform at the mother's bedside (44–47). A recent survey of the practice of using a mobile resuscitation trolley at the bedside, demonstrated that half the perinatal providers expressed concern (48). Logistical issues such as space management, and accessibility to the patient for resuscitation were reported. Larger multicenter studies (VenFirst, NCT02742454) are attempting to better answer the question about the generalizability and benefit of ventilation with an intact cord.

## Concerns related to umbilical cord milking

All available trials in human infants comparing UCM to ICC or DCC report no adverse effect of milking. Recent neurodevelopmental follow-up studies of preterm infants reported higher or similar cognitive and language scores with UCM compared to DCC at birth (29, 49). However, these trials were limited by small sample size, especially of extremely preterm infants. Recent animal data from preterm lambs demonstrates swings in carotid artery pressure and flow with umbilical cord milking (50). Extremely preterm infants (<28 weeks) have a highly vascularized germinal matrix which may be prone to bleeding if these rapid fluctuations are occurring in human infants with cord milking. The exact physiological impact of UCM on neonatal adaptation still needs more clarification. Future studies are needed to further evaluate the acute effects of cord milking on the hemodynamics in human pregnancies. The International Liaison Committee on Resuscitation (ILCOR) in 2015 stated that the long-term safety profile is still unknown, and thus recommended against the routine use of UCM in newborns <29 weeks gestation (4).

## Conclusions

Currently, some centers are using cord milking as their exclusive standard of care based on reduction in morbidities such as death and IVH after implementation of cord milking (51, 52). A recent survey of obstetricians and perinatologists in the United States reported 38.9 percent of obstetrical providers use umbilical cord milking, and in infants that need resuscitation 25 percent use umbilical cord milking (53). Thus, there is an urgent need for high quality evidence to compare the use of UCM to ICC in infants that need resuscitation. Two recent trials from India demonstrated that it is feasible to study term and preterm who are depressed at birth (54, 55). We need to determine if umbilical cord milking provides a superior placental transfusion and improves neonatal outcomes compared to current approaches. Two ongoing large multinational multicenter randomized trials (NCT03019367, NCT03631940) will provide evidence as to whether UCM is beneficial in preterm infants and infants that need resuscitation.

## Author contributions

AK wrote the initial version of the manuscript and approves the final version as it is submitted.

### Conflict of interest statement

The author declares that the research was conducted in the absence of any commercial or financial relationships that could be construed as a potential conflict of interest.
